# Skeletal Muscle Function during Exercise—Fine-Tuning of Diverse Subsystems by Nitric Oxide

**DOI:** 10.3390/ijms14047109

**Published:** 2013-03-28

**Authors:** Frank Suhr, Sebastian Gehlert, Marijke Grau, Wilhelm Bloch

**Affiliations:** Institute of Cardiovascular Research and Sport Medicine, Department of Molecular and Cellular Sport Medicine, German Sport University Cologne, Am Sportpark Müngersdorf 6, 50933 Cologne, Germany; E-Mails: suhr@dshs-koeln.de (F.S.); gehlert@dshs-koeln.de (S.G.); m.grau@dshs-koeln.de (M.G.)

**Keywords:** skeletal muscle, nitric oxide, nitric oxide synthase, cGMP, *S*-nitrosylation, exercise, signaling

## Abstract

Skeletal muscle is responsible for altered acute and chronic workload as induced by exercise. Skeletal muscle adaptations range from immediate change of contractility to structural adaptation to adjust the demanded performance capacities. These processes are regulated by mechanically and metabolically induced signaling pathways, which are more or less involved in all of these regulations. Nitric oxide is one of the central signaling molecules involved in functional and structural adaption in different cell types. It is mainly produced by nitric oxide synthases (NOS) and by non-enzymatic pathways also in skeletal muscle. The relevance of a NOS-dependent NO signaling in skeletal muscle is underlined by the differential subcellular expression of NOS1, NOS2, and NOS3, and the alteration of NO production provoked by changes of workload. In skeletal muscle, a variety of highly relevant tasks to maintain skeletal muscle integrity and proper signaling mechanisms during adaptation processes towards mechanical and metabolic stimulations are taken over by NO signaling. The NO signaling can be mediated by cGMP-dependent and -independent signaling, such as *S*-nitrosylation-dependent modulation of effector molecules involved in contractile and metabolic adaptation to exercise. In this review, we describe the most recent findings of NO signaling in skeletal muscle with a special emphasis on exercise conditions. However, to gain a more detailed understanding of the complex role of NO signaling for functional adaptation of skeletal muscle (during exercise), additional sophisticated studies are needed to provide deeper insights into NO-mediated signaling and the role of non-enzymatic-derived NO in skeletal muscle physiology.

## 1. Introduction

Skeletal muscle tissue is highly plastic and shows a wide spectrum of adaptations towards mechanical and metabolic stress, as induced by physical exercise. To achieve adaptational benefits from exercise training, a variety of skeletal muscle subsystems are involved to orchestrate the complex systemic interactions taking place in skeletal muscle tissues. This implicates at least three fundamental myocellular responses to ensure short and long-term functional integrity of skeletal muscle in response to altered demands of workload as it exists during exercise. First, the acute modulation of excitation–contraction coupling and the adapted sensitivity of contractility by reverse modification of contractile elements encourages the immediate adaptation. Second, an acute increase in substrate and oxygen uptake has to be initiated, in addition to the glycolytic and oxidative metabolic routes being turned on. Third, both acute and chronic regulation of gene expression and protein levels, as well as posttranslational modifications of proteins, are required to achieve sustainable signaling and structural adaptations of skeletal muscle to maintain and to increase exercise performance capacities.

In recent years, hypotheses emerged that a small and diffusible molecule, nitric oxide (NO), has important regulatory functions in all these cellular processes. As outlined below, NO was identified as the major vasodilator; however, NO production is not restricted to endothelial cells (ECs), but, interestingly, is produced in a variety of additional cell types, including skeletal muscles [1]. These findings are of high relevance, as they attribute the important roles of NO in physiological signaling mechanisms also to skeletal muscles.

As has been previously described, different compartments of skeletal muscle myofibers harbor divergent members of an enzyme family that mediates the formation of NO. These enzymes are known as nitric oxide synthases (NOS) and include three identified members: nNOS, iNOS, and eNOS [1]. In skeletal muscles, NOS enzymes thus contribute to acute and adaptational mechanisms via generation of nitric oxide [1]. It is well established that skeletal muscle produces NO already at rest [2]. Interestingly, however, it was observed that NO concentration increases up to 200% in response to repetitive muscle contractions [3]. Along with an increase in NO level, it was observed that reactive oxygen species (ROS) and reactive nitrogen species (RNS) formation also increase upon contractile activity [4–6]. While ROS/RNS have long been deemed deleterious for cells and tissues, possibly resulting in oxidative and nitrosative stress leading to endothelial dysfunction [7], there is now increasing evidence that both function as signaling molecules regulating vascular smooth muscle cell growth [8] and modulating changes in cell and tissue homeostasis and gene expression [9,10].

Because the underlying mechanisms and effects of NO in skeletal muscle adaptation during exercise are still elusive, we address in the following sections the most recent findings of NO signaling in skeletal muscle and place particular emphasis on exercising conditions. Furthermore, the importance of NO in skeletal muscle subsystems is highlighted with respect to exercise-induced adaptations to maintain the skeletal muscle function and structure and to improve physical performance capacities.

## 2. Basics of Nitric Oxide

Nitric oxide (NO), a member of the nitrogen oxides, is a colorless free radical that can react with other free radicals, as well as molecular oxygen (O_2_). The biological function of NO, formerly known as “endothelium-derived relaxing factor,” was discovered in mammals in the 1980s and was shown to be responsible for the regulation of vascular tone [11,12].

Subsequent studies have shown that NO is a key regulatory molecule regulating central biological processes in almost all tissues, cells and organs. NO is involved in the regulation of blood pressure through relaxation of vascular smooth muscle cells and vasodilatation of blood vessels. Impaired endothelial function as described in cardiovascular diseases, such as the coronary heart disease [13], is associated with reduced bioavailability of NO and increased generation of superoxide anions. These events restrict cyclic guanosine 3′,5′-monophosphate (cGMP)-dependent protein kinase and reduce NO/cGMP-dependent relaxation in smooth muscle cells [14]. In the immune system, NO is involved in the control of infectious diseases [15], tumors [16] and the autoimmune process [17]. In the cardiovascular system, NO limits platelet aggregation and adhesion and leads to disaggregation of already aggregated platelets [18,19]. In inflammatory processes, NO is released from the endothelium, acting in an anti-inflammatory manner by inhibiting the adhesion of leukocytes and endothelial cells. In the skeletal muscle, NO is involved in the regulation of contractility and it is also suggested that NO mediates satellite cell activation, thereby contributing to muscle repair [20].

## 3. Reaction Routes of NO

NO as a free gas exhibits a variety of reaction routes. NO either oxidizes to nitrite and nitrate [21] or reacts with O_2_ and superoxide anions to generate low molecular weight NO derivatives (NO*_x_*) [22]. Peroxynitrite, the reaction product of NO and superoxide anions, is the most reactive free radical species causing oxidative injury [23].

In the endothelium, NO is permanently produced to accommodate the increased blood flow by dilating arteries. Endothelial NO diffuses internally across the cell membrane into smooth muscle cells or into the lumen of the blood vessel, where it first reaches the blood platelets and inhibits platelet activation through activation of soluble guanylate cyclase (sGC) and cyclic guanosine 3′,5′-monophosphate (cGMP) accumulation [24]. The NO/cGMP signaling pathway also plays an important role in the cardiovascular and neural system, where it influences the relaxation of smooth muscle cells [25–27] and modulates synaptic transmission [28,29].

It is important to note that the generation of cGMP is only the first event in the signaling pathway induced by NO. cGMP is capable of activating protein kinase G (PKG) which, in turn, phosphorylates serine or threonine residues on other proteins, thereby modifying their activities [30]. Other effectors include cGMP-regulated phosphodiesterases (PDEs) and cyclic nucleotide-gated cation channels, which are involved in sensory processes, and cyclic GMP-dependent protein kinases (cGK) [26]. A key protein modulated by this action is Rho A kinase. This protein promotes the phosphorylation of myosin by activation of the myosine light chain kinase (MLCK) and inhibition of the myosin light chain phosphatase (MLCP), which opposes MLCK. cGMP-activated protein kinases enhance the inactivation of Rho A kinase by phosphorylation. This action inhibits the contraction of the smooth muscle, thus increasing blood flow which, in turn, decreases blood pressure [31]. cGMP also activates PKG1, which subsequently phosphorylates and modulates the activity of a variety of downstream targets. PKG1 decreases intracellular calcium levels in vascular smooth muscle cells following membrane hyperpolarization and inhibition of myosin light chain phosphorylation, which all contribute to vasorelaxation [32]. These mechanisms are predominantly described for smooth muscle cells. However, it is highly likely that similar pathways are involved in skeletal muscle function, although the knowledge is still highly limited. To underline the hypothesis that cGMP and mediated signaling plays a role in skeletal muscle tissue, it should be highlighted that, in a recent study, the importance of NO and cGMP in the proliferative of myogenic precursors, known as satellite cells, was described [33]. These data support the idea that NO and cGMP might overtake crucial functions in skeletal muscle, not limited to functional regulation of skeletal muscle. It remains to be elucidated whether exercise changes the signaling properties and routes of NO/cGMP-dependent events.

It is important to address that NO has also a direct mechanism of action, besides it indirect signaling via cGMP. NO interacts with free reactive cystein thiol groups of proteins to form *S*-nitrosothiols [34,35]. This reaction termed *S*-nitrosylation represents a mechanism, through which NO acts independently of the NO/cGMP pathway [36]. The binding of NO to proteins ensures, on the one hand, that the biological effectiveness of NO is preserved, since NO is a very unstable and highly reactive molecule [37]. On the other hand, the S-nitrosylation is a major posttranslational modification, which affects the functionality of proteins. S-nitrosylation is described in various tissues and cell types for a variety of proteins of different species including enzymes, membrane receptors, ion channels, transcription factors, and metalloproteins [38]. Therefore, protein activity is regulated by a precise ratio of S-nitrosylation and denitrosylation [39]. It is known that S-nitrosylations play critical roles in vascular functions, but it has not yet been determined which functions are mediated by *S*-nitrosylations in skeletal muscle functions and whether exercise has regulatory properties in the change of these signaling pathways.

## 4. Enzymatic and Non-Enzymatic Synthesis of NO

NO is biosynthesized from the amino acid l-arginine [40]. The concentration of l-arginine varies within the organism. In the blood and extracellular fluids, concentrations of 60–80 μM have been found, while cells exhibit even higher concentrations [31]. l-arginine concentrations in red blood cells, for example, have been found to be 258 ± 113 μM [41].

**Figure 1:**
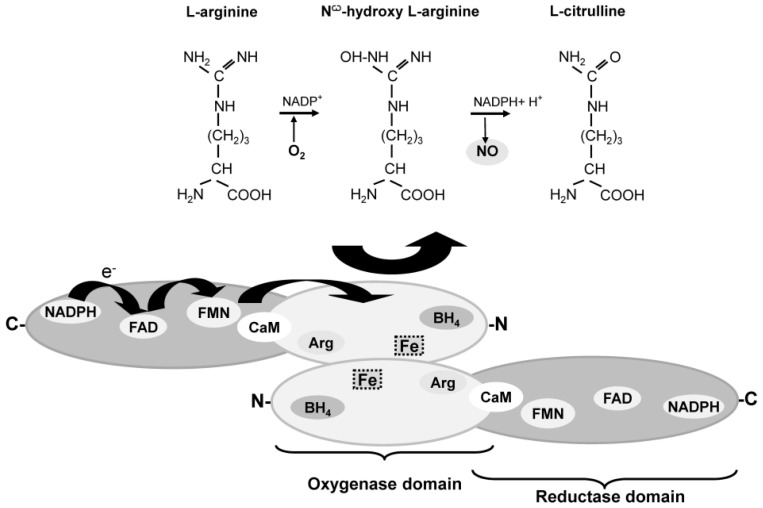
Schematic illustration of NOS dimer and NO formation from l-arginine. The two monomers are combined to form a dimer. The synthesis of NO depends on the presence of the substrate l-arginine and the cofactors including: molecular oxygen (O_2_), nicotinamide adenine dinucleotide phosphate (NADPH), flavin adenosine dinucleotide (FAD), flavin mononucleotide (FMN), calmodulin (CaM) and tetrahydrobiopterin (BH4). In reaction, l-arginine with oxygen *N*^ω^-hydroxy-l-arginine is first formed before l-citrulline and NO are produced (modified after [31]).

NO can be produced enzymatically and non-enzymatically in the cells and tissues. Enzymatic NO generation is carried out by the NOS family members. The enzymes catalyze by binding the substrate l-arginine [40], a flavin-mediated electron transfer from the electron donor nicotinamide adenine dinucleotide phosphate-oxidase (NADPH), to the prosthetic heme group. A variety of other cofactors are required for the NO formation, including O_2_, calmodulin (CaM) and tetrahydrobiopterin (BH_4_). The latter is bound near the heme group in order to transfer the electrons to a guanidino nitrogen group of l-arginine. In reaction with oxygen, *N*^ω^-hydroxy-l-arginine is first formed before l-citrulline and NO are produced [42] (Figure 1).

Although the enzymatically produced NO is mainly considered to be the major source of NO in mammals, it has long been assumed that nitrite and nitrate are harmful and/or inert oxidation products of NO [43,44]. Until recently, it was believed that both nitrate and nitrite can be recycled back to NO under acidic or highly reduced conditions which occur in disease states, such as ischemia. This NO formation is not prevented by inhibition of the NOS enzyme, and the accumulation of NO from typical nitrite concentrations found in biological tissues rises about 100-fold when the pH falls from 7.4 to 5.5 [45]. This event results in vasodilation, which implies that nitrite functions as a potent vasodilator under mild hypoxic or acidic conditions [46,47]. The vasodilation was thus thought to inversely correlate with the oxygen saturation of the hemoglobin and with the formation of iron-nitrosyl-hemoglobin and, to a lesser extent, *S*-nitrosylated hemoglobin [46]. Further studies have shown that nitrite-induced vasodilation is inhibited at high hemoglobin oxygen fractional saturation, whereas vasodilation is promoted when hemoglobin unloads to 50% saturation [48]. These processes are helpful to support the exercising skeletal muscle tissue with oxygen to maintain the physiological environment in order to produce force.

Hemoglobin, however, is not the only member of the heme globin family capable of reducing nitrite to NO under hypoxic conditions. Recently, Totzeck and colleagues [49] have shown in mice that myoglobin, the oxygen-binding protein of the skeletal and heart muscle, also reduces nitrite to NO under hypoxia [46,50,51]. Another established effect of myoglobin-dependent reduction of nitrite was the activation of the NO/sGC/cGMP signaling pathway, which leads to a reduction in blood pressure. The involvement of NOS isoforms 2 and 3 was consequently excluded [49].

## 5. Nitric Oxide Synthase (NOS) Isoforms

We have mentioned that a family of isoforms has been described. The calcium-dependent constitutive forms include neuronal NOS (nNOS, NOS1) with the mitochondrial NOS (mt-NOS) as its α-isoform [52,53]. In mature skeletal muscle and heart, an alternative splice form of nNOS, containing an insert that arises from alternative splicing of nNOS pre-RNA [54,55], has been found and termed nNOSμ (nNOSμ).

Endothelial NOS (eNOS, NOS3) [56] is another constitutively expressed NOS isoform leading to vasorelaxation [57]. Recently, the functional activity of an endothelial type NOS has been described in red blood cells (RBC-NOS) [58–60] being responsible for RBC deformability through *S*-nitrosylation of cytoskeletal proteins [61].

The inducible NOS (iNOS, NOS2) is a calcium-independent form since CaM is tightly bound and therefore no calcium increase is required for its activation. iNOS is stimulated by cytokines [62,63], such as TNF-α, IFN-γ, and IL-1β [64], which have been shown to be increased in the skeletal muscle of patients with coronary heart failure [65]. Increased iNOS activity leads to accumulation of toxic concentrations of NO leading to inhibition of key enzymes of the oxidative phosphorylation or attenuation of the contractile performance of the skeletal muscle [66]. Studies have shown that long-term endurance training reduces the local expression of IL-1β and TNF-α in quadriceps muscle [67], which also leads to a reduction in iNOS expression. This has been associated with a disinhibition of aerobic enzymes preventing the production of pro-apoptotic peroxynitrite [68]. In contrast, a study by Akita and colleagues revealed that exercise-induced increases in NO level protect the myocardium from ischemia/reperfusion injury. iNOS and eNOS have both been identified as sources for NO synthesis, but in contrast to iNOS expression, which increased only after the seventh day of exercise, eNOS activation was continuously increased. After induction of ischemia/reperfusion injury, trained mice showed significantly smaller infarct sizes, which led to the conclusion that exercise contributes to late cardioprotection against ischemia/reperfusion injury [69].

The genes for these isoforms are encoded on different chromosomes, while the genomic structure shows high similarities, indicating a common ancestral gene. nNOS is encoded on chromosome 12 (12q24.2→24.31), iNOS on chromosome 17 (17p11→17q11), and eNOS on chromosome 7 (7q35→7q36) [70,71]. The Michaelis constant (Km) of the individual isoforms were found to be 1.4–2.2 μM for the nNOS [72], 2.8, 16 and 32.3 μM for iNOS [73] and 2.9 μM for eNOS [74].

## 6. Localizations of NOS Isoforms and Their Occurrence in Skeletal Muscle Tissue

The isoforms have been named after the tissues or cells from which they have been originally purified in order of their discovery, but they are also expressed in other systems. nNOS activity was first shown in macrophages [73], but is also present in human alveolar and bronchial epithelial cells [75], carcinoma cells, vascular smooth muscle cells or endothelial cells [76]. iNOS has been found in the nervous system, in skeletal muscles and the respiratory epithelium [75] and eNOS is not only present in the epithelium, but also in the heart, skeletal muscle and in neurons [77,78].

**Figure 2:**
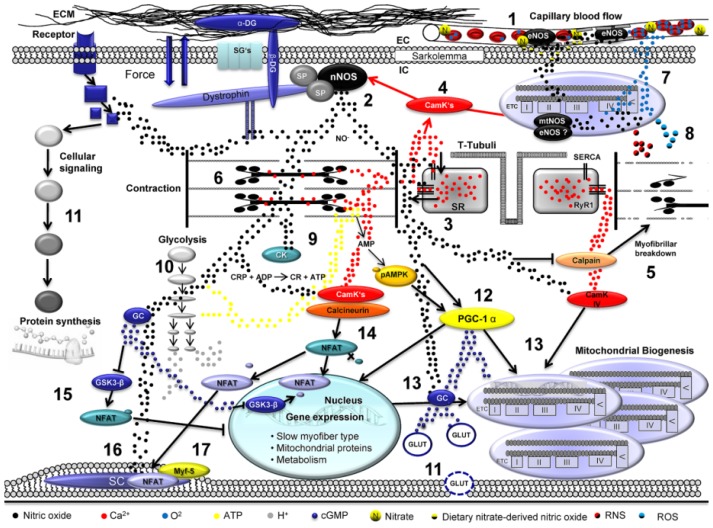
Mechanisms of nitric oxide in exercising skeletal muscle: (**1**) eNOS regulates blood flow by nitric oxide-mediated vasodilatation; (**2**) Neuronal NOS induces NO production in loaded skeletal muscle; (**3**) NO modulates RyR1 Ca^2+^ channel- and SERCA Ca^2+^ channel; (**4**) Ca^2+^ ions increase calmodulin kinase activity which also enhances NOS activity; (**5**) NO inhibits the activity of calpains which facilitate myofibrillar breakdown; (**6**) NO reversibly interacts with skeletal muscle myosin; (**7**) mtNOS/eNOS induced NO reversibly inhibits cytochrome-c oxidase (Complex IV); (**8**) NO release and oxygen turnover facilitate increased production of RNS and ROS; (**9**) NO reversibly inhibits creatinkinase and (**10**) glycolytic enzymes; (**11**) Nitric oxide increases glucose uptake via cGMP dependent GLUT translocation; (**11**) NO affects protein synthesis via modulation of cellular signaling pathways and (**12**,**13**) increases in interplay with AMPK enhanced PGC-1α activity. NO enhances the activity of calmodulin kinases also contributing to mitochondrial biogenesis; (**14**) Ca^2+^ triggers activity-induced myofiber conversions (for details see text); (**15**) This mechanism is supported by nitric oxide-mediated cGMP generation; (**16**) NO increases the proliferative activity of quiescent satellite cells in skeletal muscle (**17**) also by increased expression of Myf5 (for details see text).

The different enzymes in the organisms and their subcellular, as well as cellular localizations, lead to different functions of NO. The enzymes are therefore tailored for the locations and stimuli where NO is required. The main areas relate to the blood flow, neurotransmission and immune-response. nNOS has been detected in skeletal muscles, e.g., human gastrocnemius, omohyoideus, quadriceps, urethral sphincter and vastus lateralis muscle [79–82]. In rats and/or mouse, the diaphragm, deltoideus, extensor digitorum longus (EDL), gastrocnemius, levator labii, soleus, quadriceps, and tibialis anterior muscle have been found positive for nNOS [79,83–86]. Human skeletal muscle tissue shows higher nNOS activity than the human brain [82], while in rats, nNOS activity is higher in the brain than in the limb or diaphragm muscles [86]. nNOS enzyme has been found to bind to membranes or cytoskeletal structures [82]. Therefore, in human muscles, about 80% of nNOS activity is found in the pellet fraction compared with about 50% in mouse skeletal muscles [79], thus suggesting a tight membrane association. In contrast, in guinea pig skeletal muscle, almost all nNOS activity was found in the particulate fraction [87]. Examination of nNOS distribution in different muscle fiber types reveals that nNOS is mainly concentrated at the surface membrane of type II (fast-twitch) fibers (EDL, gastrocnemius, plantaris), whereas type I (slow-twitch; soleus) fibers shows no or only weak reaction [86,88,89]. Nevertheless, other studies by Gossrau *et al*. [90] and Kusner *et al*. [91] suggest that in rat facial muscle, and extrafusal and intrafusal fibers of sheep, sarcolemmal nNOS is present in both type I and type II fibers. Also in humans, nNOS is expressed in both muscle fiber types [90,92], although some studies state that nNOS expression is higher in the cytoplasm and at the sarcolemma of type I than type II fibers (vastus lateralis muscle) [80]. nNOS binds to α1-syntrophin, a dystrophin-associated protein (Figure 2). These three proteins are associated at the inner surface of the sarcolemma and subsarcolemmal areas near mitochondria [93]. A lack of dystrophin has been shown in patients with Duchenne muscular dystrophy and in animal models of this disease which consequently leads to a loss of nNOS from the sarcolemma.

During muscle development, nNOSμ expression parallels myotube fusion in culture but nNOS expression does not start until postnatal day 5 in human skeletal muscle. In mice, nNOS protein levels increase from week 2 to 52 [79]. In rat diaphragm, skeletal muscle showed higher activity of nNOS and eNOS in the fetal and early neonatal periods than in mature muscles, which shows declined NOS activity. Extensive nNOS expression was found at the sarcolemma in neonatal and mature diaphragms, whereas eNOS expression was limited to the endothelium [88].

iNOS is not expressed constitutively by muscle myocytes, but its expression can be induced as a response to inflammatory events [94].

eNOS shows only low levels of expression in skeletal muscle like rat EDL and soleus muscles [89]. eNOS content does not differ between type I and type II fibers and was shown to co-localize to succinate dehydrogenase—a mitochondrial marker [95,96]. In guinea pig gastrocnemius muscle, significant eNOS was detected only in the vascular endothelium where it probably contributes to the regulation of contractile response [87].

These data show interesting findings, as the NOS isoforms are tightly localized and regulated in skeletal muscle fibers. Importantly, it has to be noted that distinct differences exist regarding the precise localization of NOS isoforms in human and animal skeletal muscles [97].

Decreased NO bioavailability (due to superoxide anion mediated inactivation) or NO production is a key factor in the pathogenesis of various cardiovascular diseases, including diabetes, coronary heart disease (CHD), sepsis or atherosclerosis, and a mediator of regional circulatory disorders (ischemia/reperfusion). Xanthine oxidase, NADH/NADPH oxidase and eNOS were identified as a source of ROS/RNS generation with the latter generating ROS/RNS when the enzyme decouples in the absence of the substrate l-arginine or the NOS cofactor BH_4_[98]. Decoupling of the enzyme is also supported by peroxynitrite-mediated oxidation of BH_4_[99]. Decoupling of eNOS and the generation of oxidative/nitrosative stress with following endothelial dysfunction occurs via three mechanisms. On the one hand, the enzymatic production of NO is reduced so that the radicals, which normally react with NO, can attack cellular structures [100]. In addition, the electrons normally flowing from the reductase to the oxygenase domain are diverted to the molecular oxygen [101,102] leading to superoxide anion (O_2_^−^) generation [103]. A partial decoupling is also discussed *in vivo*. In this case, O_2_^−^ and peroxynitrite are simultaneously produced, which further increases oxidative/nitrosative stress. The external administration of NO was shown to prevent heme oxidation, inhibit the Fenton oxidation of DNA and decrease lipid peroxidation. These studies led to the conclusion that NO effectively counteracts ROS [103].

## 7. Cytoskeletal Components as Scaffold of NO Signaling

By the finding that nNOS is localized at the sarcolemma [104] (Figure 2), also in human skeletal muscle [82], the hypothesis arose that nNOS might be involved in the signaling machineries located in the skeletal muscle membrane, where mechanical forces are translated into biochemical signals to achieve certain adaptations of skeletal muscles towards mechanical impacts.

Exercise induces a mechanical stimulus on skeletal muscle fibers, where this stimulus has to be converted into a biochemical signal in order to allow skeletal muscles to adapt to the stimulus. To achieve the specific signal converting, cytoskeletal proteins are located at or in close vicinity of the sarcolemma to mediate and initiate biochemical signals. A variety of proteins mediate the conversion of mechanical impact, whereas a central cytoskeletal protein assembly in this context is known as the dystrophin-glycoprotein complex (DGC) [105]. The DGC is a complex protein assembly that is critically involved in the maintenance and integrity of skeletal muscle fibers [106]. Mutations in the DGC cause skeletal muscle dystrophies, the most prominent one is known as Duchenne muscular dystrophy (DMD) [107]. Therefore, this cytoskeletal complex takes over crucial tasks in order to translate a mechanical impact into a biochemical signal in skeletal muscles. Interestingly, it was demonstrated that the nNOS isoform is directly associated with the DGC via the protein syntrophin α1 at the sarcolemma and, thus, seems to be an important member of the DGC [108]. The disruption of the DGC–nNOS interaction can, therefore, cause skeletal muscle dystrophy showing the important role of nNOS-mediated NO formation at the sarcolemma to control skeletal muscle cytoskeletal protein functions and consequently skeletal muscle integrity. Recent research has uncovered the molecular interactions responsible for nNOS localization at the sarcolemma. Lai *et al.*[109] demonstrated by *in vivo* transfections that the spectrin-like repeats 16 and 17 (R16/17) within the rod domain of dystrophin interact with nNOS and localize it to the sarcolemma. The authors further demonstrated that treatment of mdx mice, a classical model of DMD, with a synthetic dystrophin gene containing R16/17 significantly reduced DMD-specific skeletal muscle pathology as well as increased muscle strength and exercise performance. To overcome dystrophin-null mutations and the resulting skeletal muscle dystrophies, the same group [110] unraveled the precise localization of dystrophin–nNOS interaction by substituting R16/17 by its utrophin homolog R15/16. The authors found that the α1 helix of R17 binds nNOS to the sarcolemma in coordination with flanked α2 and α3 helices, suggesting that nNOS has to be localized at the muscle membrane to exert proper functions. In contrast to these data, however, it was suggested recently that nNOS can also attenuate skeletal muscle dystrophy symptoms when failed to localize at the sarcolemma [111]. The authors used a dystrophin/utrophin double knockout model in which they expressed a muscle-specific nNOS transgene. They found that the nNOS transgene reduced dystrophy-specific pathologies, e.g. increased fibrosis, in heart, diaphragm, and hind-limb muscles. Interestingly, nNOS was not localized to the sarcolemma, suggesting that the nNOS localization to the muscle membrane is not particularly necessary to mediate beneficial adaptations in dystrophic muscles. These data highlight that nNOS failure has a mandatory function in the development of skeletal muscle dystrophies and that promising gene therapies are on their way to be developed. However, precisely which underlying nNOS-regulating mechanisms there are is still a matter of intensive debate.

In addition to skeletal muscle dystrophies, also a pathology called myasthenia gravis (MG) displays a severe skeletal muscle phenotype, and patients suffering from MG show chronic fatigue symptoms of skeletal muscles, even after initiation of appropriate immunosuppressive pharmaceutics [112]. It was demonstrated in the very recent study using an animal model for MG [113] that nNOS and, therefore, NO, play critical roles in the progression of this severe skeletal muscle disorder. The authors demonstrated that during the onset and progression of MG, the nNOS enzyme, as well as its binding partner, synthrophin α1, were lost from the sarcolemma and instead accumulated in the sarcoplasm [113]. The authors concluded from their observations that the pathological translocation of nNOS from the sarcolemma to the sarcoplasm reflects an important mechanism in the development of MG, and thus, a variety of skeletal muscle diseases.

It was speculated in the context of treating skeletal muscle diseases that the application of exercise, preferably endurance exercise, might be a promising approach to treat patients suffering from skeletal muscle diseases. The reason for this hypothesis was that it could be demonstrated that the activity of the nNOS enzyme was significantly increased after endurance exercise in rodents [3]. However, this beneficial hypothesis has to be proven in clinical trials.

Interestingly, the eNOS enzyme is also located in skeletal muscles without any skeletal muscle fiber type prevalence, though it is not located directly at the sarcolemma, but rather, is more systematically expressed in the sarcoplasm and in close vicinity of mitochondria [95] (Figure 2).

## 8. Signaling Involved in NO-Induced Modulation of Skeletal Muscle Contractility

Skeletal muscle tissue exerts a tremendous plasticity and variability regarding its functional hallmarks, including force production and fatigue resistance, both characteristics that are very important for everyday life and competitive sports. These hallmarks are achieved by skeletal muscles’ ability to rearrange their substructures and cytoarchitecture to enable adaptations to severe stimuli, such as exercise [114].

To produce the forces required for daily demands and competitive sports, the skeletal muscles initially require formations of cross-bridge formations between the sarcomeric proteins actin and myosin. This process critically involves calcium ions (Ca^2+^) that are released from skeletal muscle Ca^2+^ reservoirs, known as sarcoplasmic reticuli (SR). Subsequently, skeletal muscle contractions are primarily initiated by depolarization of the sarcolemma, a process that results in the shortening of the sarcomeres by formations of actin/myosin cross-bridges. This Ca^2+^-dependent process, known as excitation–contraction (EC) coupling, links sarcolemma depolarization to skeletal muscle contractions—a process that is dependent on NO-induced *S*-nitrosylations as outlined in the following.

Due to the localization of nNOS to the membrane-bound cytoskeleton–dystrophin complex component [115] α1-syntrophin, it can be speculated that this spatial vicinity to mechano-sensitive proteins mediates the ability to sense the transmission of forces via mechanotransduction and thus a higher mechanical load due to increased recruitment of myofibers under conditions of exercise [116]. Electromechanical coupling as the essential mechanism to induce contraction of myofibers is initiated by the rapid release of Ca^2+^ from SR. Frequent contractions lead to elevated calcium levels, thereby inducing enhanced activation of calmodulin kinase. The activity of both nNOS and eNOS is fundamentally regulated by binding to calmodulin thus enhancing NOS activity in response to exercise [117]. Hence, this mechanism constitutes a direct coupling mechanism between increased skeletal muscle loads, increased activity of NOS, and thus the release of nitric oxide. However, the contractility of skeletal muscle is itself also regulated by NO at some nodal points, possibly via redox sensitivity of target proteins.

Ca^2+^ regulation in skeletal fibers is critically regulated by a family of transmembrane Ca^2+^ release channels that are located in the membrane of the SR. These channels are ryanodine receptors (RyRs). RyRs anchor a high amount of Ca^2+^ ions at their intra-SR domain via their *C*-terminally located motif calsequestrin [118] and, thus, have important implicative roles in skeletal muscle contractions. RyRs have been shown to be modified posttranslationally by a variety of motifs, including phosphorylation or oxidations [119–121], leading to impaired functions of RyRs and consequently to reduced exercise capacity of skeletal muscles. Therefore, it seems obvious that ryanodine receptors are also prone to be posttranslationally modified by additional motifs, such as *S*-nitrosylations (Figure 2). Investigations over the last few years have discovered highly interesting findings that might be far-reaching regarding the understanding of skeletal muscle regulation and fatigue, and consequently skeletal muscle adaptations towards physical exercise.

In skeletal muscles, Ca^2+^ homeostasis is mainly regulated by a large protein complex, known as ryanodine receptor-1 (RyR1) [121,122]. It was demonstrated recently that iNOS co-localizes with RyR1 in skeletal muscles, which is why it was speculated that NOS isoforms might indirectly trigger RyR1 functions by actively produced NO [123]. Of note in this context is the observation that RyR1 possesses a cysteine residue at position 3635 (Cys3635) that is highly susceptible to be *S*-nitrosylated by NOS-formed NO [124], which is why RyR1 is a direct target of NO leading to *S*-nitrosylations of RyR1 Cys3635 residues. This posttranslational modification of RyR1 causes a conformational change of RyR1 affecting the Ca^2+^ homeostasis in skeletal muscles (Figure 2). Consequently, skeletal muscles deteriorate force production as the active contraction mechanisms are disturbed. In this context, RyR1 S-nitrosylation strengthens aging-induced sarcopenia, as uncontrolled Ca^2+^ release by RyR1 from the sarcoplasmic reticulum (SR) causes activation of Ca^2+^-dependent proteases, the calpains. Therefore, NO-mediated *S*-nitrosylation of RyR1 exhibit both acute and chronic malfunctional events in skeletal muscles, loss of contraction force, increased skeletal muscle protein breakdown, and, consequently, reduced abilities of skeletal muscles to adapt to physical exercise stimuli.

Disturbances of controlled Ca^2+^ shuffling between RyR1 and the sarcoplasm induced by *S*-nitrosylation of RyR1 cause malfunctional adaptations of skeletal muscles towards physical exercises. Therefore, taken together, these data highlight the considerable relevance of NO in the physiological handling of Ca^2+^ homeostasis in skeletal muscles. However, two questions will be important for the future to gain a detailed understanding RyR1 regulation and, thus, Ca^2+^ handling in skeletal muscles after exercise. First, it remains still unaddressed and, thus, unclear whether the type of muscle contraction induces differences in RyR1 posttranslational modifications. Second, it has to be elucidated whether also acute exercise has an impact on RyR1 biochemistry and, consequently, on Ca^2+^ homeostasis. To address the first question, we recently used a rat exercise model subjected to concentric and eccentric running conditions. Thereby, we observed that both exercise/skeletal muscle contraction conditions result in increases of RyR1 phosphorylation [125]. We also provided evidence for the second question, as we demonstrated very recently in human skeletal muscle that RyR1 phosphorylation is early and transiently increased after strenuous exercise [121]. In the context of NO-controlled Ca^2+^ homeostasis in skeletal muscle, it will be important to study *S*-nitrosylations of RyR1 after acute concentric and eccentric muscle work *in vivo* to draw a comprehensive picture of the diverse exercise-dependent mechanisms regulating Ca^2+^ homeostasis in skeletal muscles.

It is further described that other molecular targets within skeletal muscle such as the sarcoplasmic reticulum calcium ATPase (SERCA) and myosin heavy chains exhibit reversible redox sensitivity and may participate in NO modulation of excitation–contraction coupling [117]. It is documented that NOS inhibition in skeletal muscle has attenuating effects of limb muscle contractility in dogs [126] and rat diaphragm [127]. These findings suggest that endogenous NO influences cross-bridge cycling under *in vivo* situations. In skeletal muscle bundle preparations or single fiber preparations, NO administration or NOS inhibition resulted in decreased contractility [128] and force-frequency relationship [129,130], at least under submaximal power output and in isolated muscle. It is important to note that, depending on the exercise regimen, experimental conditions and, importantly, the NO concentration that is applied, unequivocal results concerning attenuated or increased contractility can be observed. A recent study by Evangelista and coworkers [131] proved a direct effect of NO via *S*-nitrosylation on myosin heavy chain isoforms in rats and human cardiac myosin heavy chain *in vitro* and *in vivo*. Interestingly, the NO action in this investigation was attributed as a molecular gear shift for myosin, as the nitrosylation state affected speed or force of myosin motor filaments with alternating effects of each of these parameters under different nitrosylation states. However, the main source of NO in all the former cases is not clear and may derive from vasculature, innervating nerves or also the muscle itself. It may be further discussed to which extent contractility of skeletal muscle preparations is influenced by experimental NO concentrations and O_2_ availability that are distinct from physiological levels [117]. Hence, the physiological correlate between contractility and NO concentration may be overestimated and rather be influenced by increased calcium sensitivity when calcium concentrations rapidly increase up to millimolar concentrations [1], especially under repeated muscle contractions. To date, the relationship between skeletal muscle contractility and NO generation under physiological conditions, especially under aspects of exercise in humans, is not clear and has yet to be investigated.

## 9. Reactive Oxygen Species/Reactive Nitrogen Species and Antioxidative Enzymes in Skeletal Muscle

ROS include the superoxide anion (O_2_^−^), hydrogen peroxide (H_2_O_2_) and the hydroxyl radicals (OH·). RNS include peroxynitrite (ONOO^−^). ROS and RNS were also determined as RNOS (reactive nitrogen oxygen species) as they also react with each. They are continuously generated in the body by an incomplete reduction of molecular oxygen and enzymatically inactivated by superoxide dismutase (SOD), catalase and glutathione peroxidase (GPX) and non-enzymatically by antioxidants (e.g., vitamins E and C, glutathione) [5]. Overproduction of RNOS can result from a variety of stressors, such as exposure to environmental pollutants [132] or physical exercise [133]. RNOS generation thus depends on the mode (aerobic, anaerobic), intensity, and duration of exercise, as varying types of exercise differ in their respective energy requirements, levels of oxygen consumption, and mechanical stresses imposed on the tissues. It has already been stated that skeletal muscle fibers produce both RNOS, in particular superoxide, and NO with every contraction, which results in the formation of secondary RNOS [4–6]. RNOS formation during exercise has been attributed to the mitochondria, potentially damaging tissues [134]. But also other cell components like the NAD(P)H oxidase enzyme associated with the sarcoplasmic reticulum [135] and the transverse tubules [136,137] within skeletal muscle have been found to produce RNOS. Recent data in contrast suggest a Janus face for RNOS. While low concentrations of RNOS modulate cell signaling processes [138] and are required for normal force production [139,140], higher RNOS concentrations reduce force production both in time- and dose-dependent manners [141] contributing to muscle fatigue. Reid *et al.*[139] aimed to explain this with a model assuming that the muscle redox state is a physiologically regulated variable balanced with matching rates of RNOS production and cellular antioxidant buffering capacity. Antioxidant enzymes including SOD, GPX, and catalase have been found in skeletal muscle. SOD dismutates superoxide radicals to form hydrogen peroxide and oxygen. 15%–35% of total SOD activity has been found in the mitochondria [142,143] with the highest activity in oxidative muscles that contain a high percentage of type I and type IIa fibers [144]. SOD activity in skeletal muscle can be modified by activity patterns with 20%–112% increases in both SOD1 and SOD2 after endurance exercise training [144,145]. Glutathione peroxidase catalyzes the reduction of hydrogen peroxide or organic hydrogen peroxide to water and alcohol using reduced glutathione as the electron donor [146,147]. The amount of GPX in skeletal muscle fibers differs across fiber types. Highly oxidative fibers (type I) contain the highest GPX activity, whereas fibers with low oxidative capacity (type IIb) possess the lowest levels of GPX (rodents) [148]. GPX is inducible and increases in skeletal muscle fibers and have been shown in regular exercise with activity increases of about 20%–177% [149,150]. Catalase catalyzes the breakdown of hydrogen peroxide into water and oxygen. The catalase protein levels are highest in oxidative muscle fibers and lowest in fibers with low oxidative capacity [143,151]. It is controversially discussed whether catalase expression increases upon exercise stimulus [152] or decreases [143].

## 10. NO-Mediated Modulation of Metabolism

In order to maintain skeletal muscle contraction abilities after the onset of exercise, the coupling between ATP demand and supply induces increased creatine phosphate breakdown via creatine kinase, as well as uptake of glucose and oxygen, to supply glycolysis and oxidative metabolism via respiratory chain complexes. All of these mechanisms can be directly modulated via NO-dependent mechanisms [1,117]. At the level of the vasculature and already at the onset of exercise, NO offers a prominent mechanism in increasing blood flow to exercising skeletal muscle. Although the impact of NO on vasodilatory effects of blood vessels is well described [153], recent work revealed a direct influence of nitric oxide on oxygen uptake kinetics in exercising skeletal muscle [154]. These authors also concluded NO to be involved in long-term improvements of oxygen uptake kinetics in exercising muscle and thus a functional role on skeletal muscle blood flow adaptations not exclusively related to acute modifications of vascular tone. Interestingly, nutritional supply of dietary nitrate has recently been shown to increase bio availability of NO under conditions of exercise [155] and resulted in decreased blood pressure and, more importantly, also to decrease oxygen cost at given workloads. Thus, besides endogenous NO production by distinct NOS isoforms located in various compartments in skeletal muscle, it is important to note that also the ingestion of NO-generating supplements affect nitric oxide-related effects in skeletal muscle.

At the level of myofiber energy metabolism NO inhibits creatine phosphate breakdown by creatine kinase. In this context some investigations [156,157] found exogenous NO donors to downregulate creatine kinase activity in striated muscle. This influences directly the ATP synthesis via CRP breakdown and thus crucially limits skeletal muscle contractility. NO donors prevented creatine phosphate depletion during periods of increased myocardial work in isolated rat heart. This creatine phosphate sparing effect was accompanied by a significant decline in intramuscular ATP levels and hence a loss of contractile function. It may be hypothesized that this effect may also serve *in vivo* as a subtle limiting factor for overreaching muscular work of skeletal muscle, since under physiological conditions, muscle contractions are terminated much earlier and before declining ATP levels may reach critical levels.

Skeletal muscle glucose uptake is meditated by several mechanisms that lead to the upregulation of GLUT4 transporters on the cell surface. Besides insulin-dependent glucose uptake via PI3K [158], also Ca^2+^[159], contraction-dependent mechanisms [160,161], or contraction-induced and AMPK phosphorylation via exercise-induced energetic stress [162] offer alternative pathways to increase glucose uptake. In an excellent study by Etgen and coworkers, it was shown that nitric oxide offers an additional important mechanism to increase glucose uptake via a cGMP-dependent pathway; however, this mechanism was independent of PI3K and Ca^2+^[163]. Thus, exercise-induced NO generation is involved in the regulation of glucose uptake under conditions of increased substrate requirements of metabolism. However, in contrast to the function of increasing glycolytic substrate availability, NO is able to reduce glycolytic flux via inhibition of glyceraldehyde-3-phosphate dehydrogenase (GAPDH) by *S*-nitrosylation of cysteine 149 [164]. However, under physiological conditions, e.g., strenuous exercise and glycogen depletion, this effect may be overridden to favor exercise-induced demands via glycolytic ATP generation [117] (Figure 2).

Importantly, nitric oxide is also capable of modulating oxidative energy metabolism via fine-tuning adjustments of the efficiency of the respiratory chain complex IV. NO competes with molecular O_2_ towards binding to the active site of cytochrome c oxidase, thereby inhibiting its activity [165] (Figure 2). Depending on the concentration of NO, this mechanism is highly reversible; however, it takes place under physiological conditions very quickly after the onset of exercise. There is discussion that this inhibition of complex IV modulates mitochondrial respiration rather than inhibits it [166]. This is believed to occur insofar as cyotochrome c oxidase molecules that are inhibited by NO under physiological conditions may preserve redox state and increase sensitivity of remaining and unmodified cytochrome c oxidase proteins under conditions of declined O_2_ partial pressure and changed metabolic environment. This mechanism may thus act as a subtle and quickly responding modulator of oxidative metabolism to ensure integrity of metabolism under conditions of exercise and hypoxia.

## 11. NO Signaling in Skeletal Muscle Hypertrophy

Beside Ca^2+^ homeostasis, exercise-induced hypertrophy is an important hallmark of skeletal muscles to adapt to increased loading and to produce higher forces to sustain daily demands and competitive sports. Skeletal muscle hypertrophy is regulated by a variety of different signaling cascades, of which the insulin-like growth factor-1/mechano growth factor/protein kinase B/mammalian target of rapamycin (Igf-1/MGF/PKB/mTOR) axis is one of the most important and thus best-characterized pathways [167].

Among the variety of skeletal muscle hypertrophic signaling routes with the Igf-1/MGF/PKB/mTOR axis in the center, it became clear in recent years that signaling pathways mediated by nitric oxide synthases-generated NO have important roles in the regulation of skeletal muscle hypertrophy. It is known that an inhibition of NOS activities results in decreases of skeletal muscle hypertrophy and, thus, skeletal muscle loss during aging. However, the underlying mechanisms are still only incompletely understood. Using the specific NOS inhibitors *N*(G)-nitro-l-arginine methyl ester (l-NAME) and 1-(2-trifluoromethyl-pheny)-imidazol (TRIM), it was demonstrated in a chronic overload rat model that skeletal muscle hypertrophy is induced by TRIM treatment by inductions of Igf-1 and its splicing isoform mechano growth factor (MGF). Furthermore, the authors found an increased ratio of phosphorylated-to-total p70S6 kinase, which is a major downstream effector of mTOR in skeletal muscle hypertrophy signaling [168]. Therefore, it can be hypothesized that NO derived from NOS enzymes provides a negative feedback control of the Igf-1/MGF/PKB/mTOR signaling axis to keep unrestricted skeletal muscle hypertrophy under control (Figure 2).

Skeletal muscle training is an effective strategy to counteract age-related loss of muscle mass, known as sarcopenia. It was demonstrated that during sarcopenia the nNOS dislocates from skeletal muscle cytoskeletal elements (see below), which is directly associated with reduced muscle fiber size, strength, and function [169]. By pharmacologically substituting the NO donor isosorbide dinitrate to old mice, it was found that the quadriceps muscle mass was increased by 25% in combination with running exercise [169]. These data show that the stimulation of NOS isoforms combined with regular endurance exercise training owns a profound potential to counteract sarcopenia-related loss of skeletal muscle mass.

Most recently, a new pathway exploring the mechanisms of NO-induced skeletal muscle hypertrophy was described [170]. The pathway connects Ca^2+^ shuffling and skeletal muscle hypertrophic adaptations. Ito *et al.*[170] demonstrated that nNOS regulates load-induced skeletal muscle hypertrophy by activating the transient receptor potential cation channel member 1 (TRPV1). The authors precisely dissect the molecular interactions by using nNOS-null mice, in which the load-induced skeletal muscle hypertrophy was prevented. The authors describe that a mechanical load activates nNOS at the sarcolemma, which subsequently results in NO formation. NO reacts with superoxide anion to a more stable derivate, known as peroxynitrite (ONOO^−^) that in turn activates TRPV1. The release of Ca^2+^ triggers the activation of mTOR, which has demonstrably a positive effect on skeletal muscle hypertrophy.

Another important skeletal muscle-related field of research is aging-related mechanisms. During aging the skeletal muscle mass is progressively reduced, a phenomenon known as sarcopenia. It was suggested that the protein breakdown rate, especially of myofibril proteins, is highly increased during aging directly, leading to sarcopenia. However, the underlying mechanisms are far from being resolved. Recently, Samengo *et al.*[171] observed that calpains, a family of Ca^2+^-dependent proteases, are involved in these processes. Interestingly, the authors found that calpains are posttranslationally modified by NO resulting in *S*-nitrosylations thereby inhibiting calpain activity and myofibrillar protein breakdown. However, during aging, the nNOS expression is reduced in skeletal muscles directly judging decreased NO-mediated *S*-nitrosylation of calpains [171]. As a result, the myofibrillar protein breakdown is increased during aging. These data shed new light on an additional important regulatory pathway of protein turnover in skeletal muscles specifically mediated by skeletal muscle-derived NO.

Together, these recent data demonstrate that nitric oxide has a pivotal role in the regulation of exercise-induced skeletal muscle hypertrophic events, as well as during aging of skeletal muscle tissue. Although future studies are needed to carefully study the underlying mechanisms, the pharmacological stimulation of NOS enzymes to produce nitric oxide in skeletal muscles seems to be a promising strategy to counteract age-related loss of muscle mass or to increase muscle mass after injury in competitive athletes.

## 12. NO and NO-Dependent Signaling in Satellite Cells

Skeletal muscle adaptations, such as skeletal muscle hypertrophy, are importantly regulated and induced by the activation of a population of adult muscle stem cells, which are known as satellite cells. Satellite cells are resident myogenic stem cells that also play central roles in skeletal muscle regeneration processes, as induced by intensive exercise training, which is why these cells have been studied extensively in recent years to better understand the high plasticity of skeletal muscle tissue.

Hypertrophic phenotypes of skeletal muscles, also present during regeneration processes, are complex operations, because the skeletal muscle fibers have to guarantee an efficient support of the hypertophic or regenerating areas. Therefore, the maintenance area covered by one nucleus within the skeletal muscle fiber has to be optimal to guarantee an efficient support. The maintenance area covered by one nucleus is called myonuclear domain [172,173]. To achieve an optimal size of the myonuclear domains, skeletal muscles possess the ability to activate satellite cells to fuse with adult hypertrophic or regenerating skeletal muscle fibers [174,175]. Therefore, this process has important implications for skeletal muscle plasticity and adaptations. The interesting question thus arises whether NO and NO-dependent signaling pathways might also be involved in the activation and regulation of satellite cells.

In a recent *in vitro* study, it was observed that NO possesses a key role in the maintenance of skeletal muscle precursors. The authors showed that NO stimulated satellite cell proliferation by a pathway dependent on cGMP generation [33]. Furthermore, this signaling pathway contributed to the activation of satellite cell asymmetric self-renewal abilities, leading the authors to conclude that NO-dependent satellite cell activation contributes to the maintenance of a functional satellite cell pool in skeletal muscle tissue [33]. Nitric oxide further mechanistically affects satellite cell proliferation via NFAT-dependent mechanisms. It has been shown by Martins *et al.*[176] that NO directly modulates NFAT phosphorylation states and thereby its nuclear abundance. Interestingly, a satellite cell marker and regulator, known as Mfy5 [177], is also regulated by NFAT, thus indicating important roles of this protein also in skeletal muscle regenerative mechanisms [178]. Therefore, by combining these results, it seems to be obvious that nitric oxide also exerts positive effects on satellite cell regulation by stimulating and regulating the calcineurin/NFAT pathway, which is why NO represents a key signaling molecule in skeletal muscle regenerative medicine.

However, the precise underlying mechanisms of NO-mediated satellite cell regulation are still only incompletely understood and remain to be investigated carefully by future studies in order to clarify the role of NO and NO-related signaling pathways in satellite cell-dependent skeletal muscle plasticity in *in vivo* conditions.

## 13. NO-Induced Modulation of Exercise-Induced Skeletal Muscle Metabolic Demands and Myofiber Type Conversions

Nitric oxide offers functional roles in skeletal muscle adaptation. As has been described, NO interacts with components of the IGF1/MGF/AKT/mTOR axis and influences cellular signaling to skeletal muscle protein synthesis. A further important mechanism in which nitric oxide is involved affects the adaptation of oxidative metabolism via mitochondrial biogenesis [179]. The induction of mitochondrial biogenesis occurs e.g., in response to mild caloric restriction [180] but is fundamentally increased as a cellular response to endurance exercise [181]. Mitochondrial biogenesis requires a complex interplay of cellular stressors [182] that induce increased ROS production [9,183], 5′-AMP-activated protein kinase (AMPK) phosphorylation [184] and MAP kinase signaling [185] to increase the expression of mitochondrial transcription factors and importantly peroxisome proliferator-activated receptor-γ coactivator 1α (PGC-1α). PGC-1α is a crucial factor for mitochondrial biogenenesis and further offers important functions in energy metabolism [182,186]. Recent studies showed that NO together with AMPK affects the increased expression of PGC-1α in mice. This effect is mediated by increased phosphorylation of AMPK at the α1 subunit which increases endogenous NO production. In the light of the aforementioned aspects of NO on skeletal muscle contractility, this mechanism may thus serve as additional metabolically driven feedback for skeletal muscle NO generation by exercise-induced energetic stress, since AMPK activity is considerably increased in response to increased AMP levels upon exercise [187] (Figure 2). Despite AMPK-dependent signaling, it has been recently shown that calcium-induced increases in mitochondrial biogenesis via enhanced activation of calmodulin kinase can be attenuated via experimental inhibition of NOS isoforms in L6 myotubes [188]. However, under *in vivo* conditions, the picture deriving from NO-supported mitochondrial biogenesis is not completely understood. Wadley and colleagues showed recently that the involvement of endogenous nitric oxide in mitochondrial biogenesis is important for the modulation of basal but not exercise-induced biogenesis [189]. In general, the NO-dependent actions that support mitochondrial biogenesis are mediated by cGMP-dependent mechanisms [179,190].

For the exercising skeletal muscle, increased expression of PGC-1α has fundamental effects for long-term structural adaptation of skeletal muscle in the form of increased mitochondrial density and changing myofiber distribution. In the latter case it has been shown that the expression of PGC-1α contributes to increased expression of proteins that are involved in coding a slow myofiber phenotype [191]. Through this mechanism, NO can contribute to skeletal myofiber conversions that can be observed in response to continued endurance exercise [192]. The mechanism of myofiber conversion is substantially driven by changes in the expression pattern of slow genes as a direct result of continued neuromuscular activity of myofibers [193]. The resulting elevations in sarcoplasmic Ca^2+^ levels induce increases in calcineurin activity which triggers NFAT dephosphorylation and consequently the most important mechanism in activity-induced slow myofiber type conversion [194]. Recent work demonstrated that the experimental inhibition of NOS prevented the conversion of myofibers to a slow phenotype [176,195]. These events are further supported by Ca^2+^-induced activations of NOS proteins that in turn lead to cGMP production by which GSK-3β is inhibited and therefore NFAT abundance is strengthened in the nucleus [195]. This reveals in the long term the importance of NO for activity-dependent adaptations of skeletal muscle myofiber types on a structural level.

For the exercising skeletal muscle, both mitochondrial density and myofiber types are important determinants of exercise capacity. Coupled with increased mitochondrial biogenesis via NO-supported increase in PGC-1α expression is the concomitant upregulation of the mitochondrial ROS detoxification system [166] (Figure 2). This ensures the ability of skeletal muscle to deal with increased oxygen flux in skeletal muscle which is associated with increased generation of ROS molecules, especially under conditions of higher oxidative exercise capacities. Interestingly, differences in short and long-term NO application mediate either down- or upregulation of PGC-1α expression, which gives rise to divergent effects of skeletal muscle adaptation towards long-term preconditioned skeletal muscle [166].

Taken together, these data demonstrate that NO, primarily generated by nNOS in skeletal muscle tissue, takes over a variety of highly relevant tasks to maintain skeletal muscle integrity and proper signaling mechanisms during adaptational processes towards mechanical and metabolic stimulations. With the finding that especially endurance exercise training exerts beneficial outcomes in the activation of the nNOS enzyme, it will be interesting for future studies to focus on the precise underlying mechanisms that might determine skeletal muscle adaptations in pathological circumstances towards physical exercise.

## 14. Conclusions

Taken together, these data demonstrate that NO, primarily generated by NOS1 in skeletal muscle tissue, takes over a variety of highly relevant tasks to maintain skeletal muscle integrity and proper signaling mechanisms during adaptational processes towards mechanical and metabolic stimulations, such as exercise. Through the finding that especially endurance exercise training exerts beneficial outcomes in the activation of the NOS1 enzyme, it will be interesting for future studies to focus on the precise underlying mechanisms that might determine skeletal muscle adaptations in pathological circumstances towards physical exercise. Additionally, the role of NO produced by NOS2 and NOS3, as well as the non-enzymatic generating pathways for functional and structural adaptation to physical exercise, under physiological and pathophysiological conditions need to be better understood in order to gain a comprehensive picture of NO and NO-dependent signaling in skeletal muscles.

## References

[1] Stamler J.S., Meissner G. (2001). Physiology of nitric oxide in skeletal muscle. Physiol. Rev.

[2] Reid M.B., Haack K.E., Franchek K.M., Valberg P.A., Kobzik L., West M.S. (1992). Reactive oxygen in skeletal muscle. I. Intracellular oxidant kinetics and fatigue *in vitro*. J. Appl. Physiol.

[3] Balon T.W., Nadler J.L. (1994). Nitric oxide release is present from incubated skeletal muscle preparations. J. Appl. Physiol.

[4] Palomero J., Pye D., Kabayo T., Spiller D.G., Jackson M.J. (2008). *In situ* detection and measurement of intracellular reactive oxygen species in single isolated mature skeletal muscle fibers by real time fluorescence microscopy. Antioxid. Redox. Signal.

[5] Powers S.K., Jackson M.J. (2008). Exercise-induced oxidative stress: Cellular mechanisms and impact on muscle force production. Physiol. Rev.

[6] Pye D., Palomero J., Kabayo T., Jackson M.J. (2007). Real-time measurement of nitric oxide in single mature mouse skeletal muscle fibres during contractions. J. Physiol.

[7] Molavi B., Mehta J.L. (2004). Oxidative stress in cardiovascular disease: Molecular basis of its deleterious effects, its detection, and therapeutic considerations. Curr. Opin. Cardiol.

[8] Rao G.N., Berk B.C. (1992). Active oxygen species stimulate vascular smooth muscle cell growth and proto-oncogene expression. Circ. Res.

[9] Droge W. (2002). Free radicals in the physiological control of cell function. Physiol. Rev.

[10] Jackson M.J., Papa S., Bolanos J., Bruckdorfer R., Carlsen H., Elliott R.M., Flier J., Griffiths H.R., Heales S., Holst B. (2002). Antioxidants, reactive oxygen and nitrogen species, gene induction and mitochondrial function. Mol. Aspects Med.

[11] Furchgott R.F., Zawadzki J.V. (1980). The obligatory role of endothelial cells in the relaxation of arterial smooth muscle by acetylcholine. Nature.

[12] Palmer R.M., Ferrige A.G., Moncada S. (1987). Nitric oxide release accounts for the biological activity of endothelium-derived relaxing factor. Nature.

[13] Anderson T.J., Uehata A., Gerhard M.D., Meredith I.T., Knab S., Delagrange D., Lieberman E.H., Ganz P., Creager M.A., Yeung A.C. (1995). Close relation of endothelial function in the human coronary and peripheral circulations. J. Am. Coll. Cardiol.

[14] Oelze M., Mollnau H., Hoffmann N., Warnholtz A., Bodenschatz M., Smolenski A., Walter U., Skatchkov M., Meinertz T., Munzel T. (2000). Vasodilator-stimulated phosphoprotein serine 239 phosphorylation as a sensitive monitor of defective nitric oxide/cGMP signaling and endothelial dysfunction. Circ. Res.

[15] Nathan C., Shiloh M.U. (2000). Reactive oxygen and nitrogen intermediates in the relationship between mammalian hosts and microbial pathogens. Proc. Natl. Acad. Sci. USA.

[16] Pervin S., Singh R., Chaudhuri G. (2001). Nitric oxide-induced cytostasis and cell cycle arrest of a human breast cancer cell line (MDA-MB-231): Potential role of cyclin D1. Proc. Natl. Acad. Sci. USA.

[17] Kolb H., Kolb-Bachofen V. (1998). Nitric oxide in autoimmune disease: Cytotoxic or regulatory mediator?. Immunol. Today.

[18] Stamler J., Mendelsohn M.E., Amarante P., Smick D., Andon N., Davies P.F., Cooke J.P., Loscalzo J. (1989). *N*-acetylcysteine potentiates platelet inhibition by endothelium-derived relaxing factor. Circ. Res.

[19] Radomski M.W., Palmer R.M., Moncada S. (1987). The anti-aggregating properties of vascular endothelium: Interactions between prostacyclin and nitric oxide. Br. J. Pharmacol.

[20] Anderson J.E. (2000). A role for nitric oxide in muscle repair: Nitric oxide-mediated activation of muscle satellite cells. Mol. Biol. Cell.

[21] Hendgen-Cotta U., Grau M., Rassaf T., Gharini P., Kelm M., Kleinbongard P. (2008). Reductive gas-phase chemiluminescence and flow injection analysis for measurement of the nitric oxide pool in biological matrices. Methods Enzymol.

[22] Stamler J.S., Singel D.J., Loscalzo J. (1992). Biochemistry of nitric oxide and its redox-activated forms. Science.

[23] Freeman B. (1994). Free radical chemistry of nitric oxide. Looking at the dark side. Chest.

[24] Mellion B.T., Ignarro L.J., Ohlstein E.H., Pontecorvo E.G., Hyman A.L., Kadowitz P.J. (1981). Evidence for the inhibitory role of guanosine 3′,5′-monophosphate in ADP-induced human platelet aggregation in the presence of nitric oxide and related vasodilators. Blood.

[25] Lincoln T.M. (1989). Cyclic GMP and mechanisms of vasodilation. Pharmacol. Ther.

[26] Lincoln T.M., Dey N., Sellak H. (2001). Invited review: cGMP-dependent protein kinase signaling mechanisms in smooth muscle: From the regulation of tone to gene expression. J. Appl. Physiol.

[27] Hofmann F., Ammendola A., Schlossmann J. (2000). Rising behind NO: cGMP-dependent protein kinases. J. Cell Sci.

[28] Garthwaite J., Charles S.L., Chess-Williams R. (1988). Endothelium-derived relaxing factor release on activation of NMDA receptors suggests role as intercellular messenger in the brain. Nature.

[29] Zhuo M., Hawkins R.D. (1995). Long-term depression: A learning-related type of synaptic plasticity in the mammalian central nervous system. Rev. Neurosci.

[30] Munzel T., Feil R., Mulsch A., Lohmann S.M., Hofmann F., Walter U. (2003). Physiology and pathophysiology of vascular signaling controlled by guanosine 3′,5′-cyclic monophosphate-dependent protein kinase [corrected]. Circulation.

[31] Bruckdorfer R. (2005). The basics about nitric oxide. Mol. Aspects Med.

[32] Thoonen R., Sips P.Y., Bloch K.D., Buys E.S. (2013). Pathophysiology of hypertension in the absence of nitric oxide/cyclic GMP signaling. Curr. Hypertens. Rep.

[33] Buono R., Vantaggiato C., Pisa V., Azzoni E., Bassi M.T., Brunelli S., Sciorati C., Clementi E. (2012). Nitric oxide sustains long-term skeletal muscle regeneration by regulating fate of satellite cells via signaling pathways requiring Vangl2 and cyclic GMP. Stem Cells.

[34] Foster M.W., McMahon T.J., Stamler J.S. (2003). *S*-nitrosylation in health and disease. Trends Mol. Med.

[35] Martinez-Ruiz A., Lamas S. (2004). *S*-nitrosylation: A potential new paradigm in signal transduction. Cardiovasc. Res.

[36] Broillet M.C. (1999). *S*-nitrosylation of proteins. Cell Mol. Life Sci.

[37] Stamler J.S., Simon D.I., Osborne J.A., Mullins M.E., Jaraki O., Michel T., Singel D.J., Loscalzo J. (1992). *S*-nitrosylation of proteins with nitric oxide: Synthesis and characterization of biologically active compounds. Proc. Natl. Acad. Sci. USA.

[38] Hess D.T., Matsumoto A., Kim S.O., Marshall H.E., Stamler J.S. (2005). Protein *S*-nitrosylation: purview and parameters. Nat. Rev. Mol. Cell Biol.

[39] Lima B., Forrester M.T., Hess D.T., Stamler J.S. (2010). *S*-nitrosylation in cardiovascular signaling. Circ. Res.

[40] Palmer R.M., Rees D.D., Ashton D.S., Moncada S. (1988). L-arginine is the physiological precursor for the formation of nitric oxide in endothelium-dependent relaxation. Biochem. Biophys. Res. Commun.

[41] Filho J.C., Bergstrom J., Stehle P., Furst P. (1997). Simultaneous measurements of free amino acid patterns of plasma, muscle and erythrocytes in healthy human subjects. Clin Nutr.

[42] Stuehr D.J., Kwon N.S., Nathan C.F., Griffith O.W., Feldman P.L., Wiseman J. (1991). *N* omega-hydroxy-L-arginine is an intermediate in the biosynthesis of nitric oxide from L-arginine. J. Biol. Chem.

[43] Spiegelhalder B., Eisenbrand G., Preussmann R. (1976). Influence of dietary nitrate on nitrite content of human saliva: Possible relevance to *in vivo* formation of *N*-nitroso compounds. Food Cosmet. Toxicol.

[44] Tannenbaum S.R., Correa P. (1985). Nitrate and gastric cancer risks. Nature.

[45] Zweier J.L., Samouilov A., Kuppusamy P. (1999). Non-enzymatic nitric oxide synthesis in biological systems. Biochim. Biophys. Acta.

[46] Cosby K., Partovi K.S., Crawford J.H., Patel R.P., Reiter C.D., Martyr S., Yang B.K., Waclawiw M.A., Zalos G., Xu X. (2003). Nitrite reduction to nitric oxide by deoxyhemoglobin vasodilates the human circulation. Nat. Med.

[47] Gladwin M.T., Shelhamer J.H., Schechter A.N., Pease-Fye M.E., Waclawiw M.A., Panza J.A., Ognibene F.P., Cannon R.O. (2000). Role of circulating nitrite and *S*-nitrosohemoglobin in the regulation of regional blood flow in humans. Proc. Natl. Acad. Sci. USA.

[48] Isbell T.S., Gladwin M.T., Patel R.P. (2007). Hemoglobin oxygen fractional saturation regulates nitrite-dependent vasodilation of aortic ring bioassays. Am. J. Physiol. Heart Circ. Physiol.

[49] Totzeck M., Hendgen-Cotta U.B., Luedike P., Berenbrink M., Klare J.P., Steinhoff H.J., Semmler D., Shiva S., Williams D., Kipar A. (2012). Nitrite regulates hypoxic vasodilation via myoglobin-dependent nitric oxide generation. Circulation.

[50] Shiva S., Huang Z., Grubina R., Sun J., Ringwood L.A., MacArthur P.H., Xu X., Murphy E., Darley-Usmar V.M., Gladwin M.T. (2007). Deoxymyoglobin is a nitrite reductase that generates nitric oxide and regulates mitochondrial respiration. Circ. Res.

[51] Rassaf T., Flogel U., Drexhage C., Hendgen-Cotta U., Kelm M., Schrader J. (2007). Nitrite reductase function of deoxymyoglobin: oxygen sensor and regulator of cardiac energetics and function. Circ. Res.

[52] Haynes V., Elfering S., Traaseth N., Giulivi C. (2004). Mitochondrial nitric-oxide synthase: Enzyme expression, characterization, and regulation. J. Bioenerg. Biomembr.

[53] Giulivi C., Poderoso J.J., Boveris A. (1998). Production of nitric oxide by mitochondria. J. Biol. Chem.

[54] Brenman J.E., Xia H., Chao D.S., Black S.M., Bredt D.S. (1997). Regulation of neuronal nitric oxide synthase through alternative transcripts. Dev. Neurosci.

[55] Silvagno F., Xia H., Bredt D.S. (1996). Neuronal nitric-oxide synthase-mu, an alternatively spliced isoform expressed in differentiated skeletal muscle. J. Biol. Chem.

[56] Amancharla M.R., Rodarte J.R., Boriek A.M. (2001). Modeling the kinematics of the canine midcostal diaphragm. Am. J. Physiol. Regul. Integr. Comp. Physiol.

[57] Ignarro L.J. (1989). Heme-dependent activation of soluble guanylate cyclase by nitric oxide: Regulation of enzyme activity by porphyrins and metalloporphyrins. Semin. Hematol.

[58] Kleinbongard P., Schulz R., Rassaf T., Lauer T., Dejam A., Jax T., Kumara I., Gharini P., Kabanova S., Ozuyaman B. (2006). Red blood cells express a functional endothelial nitric oxide synthase. Blood.

[59] Cortese-Krott M.M., Rodriguez-Mateos A., Sansone R., Kuhnle G.G., Thasian-Sivarajah S., Krenz T., Horn P., Krisp C., Wolters D., Heiss C. (2012). Human red blood cells at work: Identification and visualization of erythrocytic eNOS activity in health and disease. Blood.

[60] Suhr F., Brenig J., Muller R., Behrens H., Bloch W., Grau M. (2012). Moderate exercise promotes human RBC-NOS activity, NO production and deformability through Akt kinase pathway. PLoS One.

[61] Grau M., Pauly S., Ali J., Walpurgis K., Thevis M., Bloch W., Suhr F. (2013). RBC-NOS-dependent *S*-nitrosylation of cytoskeletal proteins improves RBC dformability. PLoS One.

[62] Cho H.J., Xie Q.W., Calaycay J., Mumford R.A., Swiderek K.M., Lee T.D., Nathan C. (1992). Calmodulin is a subunit of nitric oxide synthase from macrophages. J. Exp. Med.

[63] Nathan C. (1997). Inducible nitric oxide synthase: What difference does it make?. J. Clin. Invest.

[64] Forstermann U., Closs E.I., Pollock J.S., Nakane M., Schwarz P., Gath I., Kleinert H. (1994). Nitric oxide synthase isozymes. Characterization, purification, molecular cloning, and functions. Hypertension.

[65] Adams V., Yu J., Mobius-Winkler S., Linke A., Weigl C., Hilbrich L., Schuler G., Hambrecht R. (1997). Increased inducible nitric oxide synthase in skeletal muscle biopsies from patients with chronic heart failure. Biochem. Mol. Med.

[66] Ungureanu-Longrois D., Balligand J.L., Kelly R.A., Smith T.W. (1995). Myocardial contractile dysfunction in the systemic inflammatory response syndrome: Role of a cytokine-inducible nitric oxide synthase in cardiac myocytes. J. Mol. Cell Cardiol.

[67] Gielen S., Adams V., Linke A., Erbs S., Mobius-Winkler S., Schubert A., Schuler G., Hambrecht R. (2005). Exercise training in chronic heart failure: Correlation between reduced local inflammation and improved oxidative capacity in the skeletal muscle. Eur. J. Cardiovasc. Prev. Rehabil.

[68] Schulze P.C., Gielen S., Schuler G., Hambrecht R. (2002). Chronic heart failure and skeletal muscle catabolism: Effects of exercise training. Int. J. Cardiol.

[69] Akita Y., Otani H., Matsuhisa S., Kyoi S., Enoki C., Hattori R., Imamura H., Kamihata H., Kimura Y., Iwasaka T. (2007). Exercise-induced activation of cardiac sympathetic nerve triggers cardioprotection via redox-sensitive activation of eNOS and upregulation of iNOS. Am. J. Physiol. Heart Circ. Physiol.

[70] Xu W., Charles I.G., Moncada S., Gorman P., Sheer D., Liu L., Emson P. (1994). Mapping of the genes encoding human inducible and endothelial nitric oxide synthase (NOS2 and NOS3) to the pericentric region of chromosome 17 and to chromosome 7, respectively. Genomics.

[71] Alderton W.K., Cooper C.E., Knowles R.G. (2001). Nitric oxide synthases: structure, function and inhibition. Biochem. J.

[72] Bredt D.S., Snyder S.H. (1990). Isolation of nitric oxide synthetase, a calmodulin-requiring enzyme. Proc. Natl. Acad. Sci. USA.

[73] Yui Y., Hattori R., Kosuga K., Eizawa H., Hiki K., Kawai C. (1991). Purification of nitric oxide synthase from rat macrophages. J. Biol. Chem.

[74] Pollock J.S., Forstermann U., Mitchell J.A., Warner T.D., Schmidt H.H., Nakane M., Murad F. (1991). Purification and characterization of particulate endothelium-derived relaxing factor synthase from cultured and native bovine aortic endothelial cells. Proc. Natl. Acad. Sci. USA.

[75] Asano K., Chee C.B., Gaston B., Lilly C.M., Gerard C., Drazen J.M., Stamler J.S. (1994). Constitutive and inducible nitric oxide synthase gene expression, regulation, and activity in human lung epithelial cells. Proc. Natl. Acad. Sci. USA.

[76] Nathan C. (1992). Nitric oxide as a secretory product of mammalian cells. FASEB J.

[77] Snyder L.M., Fortier N.L., Trainor J., Jacobs J., Leb L., Lubin B., Chiu D., Shohet S., Mohandas N. (1985). Effect of hydrogen peroxide exposure on normal human erythrocyte deformability, morphology, surface characteristics, and spectrin-hemoglobin cross-linking. J. Clin. Invest.

[78] Shaul P.W. (2002). Regulation of endothelial nitric oxide synthase: Location, location, location. Annu. Rev. Physiol.

[79] Chang W.J., Iannaccone S.T., Lau K.S., Masters B.S., McCabe T.J., McMillan K., Padre R.C., Spencer M.J., Tidball J.G., Stull J.T. (1996). Neuronal nitric oxide synthase and dystrophin-deficient muscular dystrophy. Proc. Natl. Acad. Sci. USA.

[80] Frandsen U., Lopez-Figueroa M., Hellsten Y. (1996). Localization of nitric oxide synthase in human skeletal muscle. Biochem. Biophys. Res. Commun.

[81] Ho K.M., McMurray G., Brading A.F., Noble J.G., Ny L., Andersson K.E. (1998). Nitric oxide synthase in the heterogeneous population of intramural striated muscle fibres of the human membranous urethral sphincter. J. Urol.

[82] Nakane M., Schmidt H.H., Pollock J.S., Forstermann U., Murad F. (1993). Cloned human brain nitric oxide synthase is highly expressed in skeletal muscle. FEBS Lett.

[83] Blottner D., Luck G. (1998). Nitric oxide synthase (NOS) in mouse skeletal muscle development and differentiated myoblasts. Cell Tissue Res.

[84] Christova T., Grozdanovic Z., Gossrau R. (1997). Nitric oxide synthase (NOS) I during postnatal development in rat and mouse skeletal muscle. Acta Histochem.

[85] Guo Y., Greenwood M.T., Petrof B.J., Hussain S.N. (1999). Expression and regulation of protein inhibitor of neuronal nitric oxide synthase in ventilatory muscles. Am. J. Respir. Cell Mol. Biol.

[86] Kobzik L., Reid M.B., Bredt D.S., Stamler J.S. (1994). Nitric oxide in skeletal muscle. Nature.

[87] Gath I., Closs E.I., Godtel-Armbrust U., Schmitt S., Nakane M., Wessler I., Forstermann U. (1996). Inducible NO synthase II and neuronal NO synthase I are constitutively expressed in different structures of guinea pig skeletal muscle: Implications for contractile function. FASEB J.

[88] El D.Q., Guo Y., Comtois A., Zhu E., Greenwood M.T., Bredt D.S., Hussain S.N. (1998). Ontogenesis of nitric oxide synthases in the ventilatory muscles. Am. J. Respir. Cell Mol. Biol.

[89] Kapur S., Bedard S., Marcotte B., Cote C.H., Marette A. (1997). Expression of nitric oxide synthase in skeletal muscle: A novel role for nitric oxide as a modulator of insulin action. Diabetes.

[90] Gossrau R. (1998). Caveolin-3 and nitric oxide synthase I in healthy and diseased skeletal muscle. Acta Histochem.

[91] Kusner L.L., Kaminski H.J. (1996). Nitric oxide synthase is concentrated at the skeletal muscle endplate. Brain Res.

[92] Grozdanovic Z., Gosztonyi G., Gossrau R. (1996). Nitric oxide synthase I (NOS-I) is deficient in the sarcolemma of striated muscle fibers in patients with Duchenne muscular dystrophy, suggesting an association with dystrophin. Acta Histochem.

[93] Wakayama Y., Inoue M., Murahashi M., Shibuya S., Jimi T., Kojima H., Oniki H. (1997). Ultrastructural localization of alpha 1-syntrophin and neuronal nitric oxide synthase in normal skeletal myofiber, and their relation to each other and to dystrophin. Acta Neuropathol.

[94] Williams G., Brown T., Becker L., Prager M., Giroir B.P. (1994). Cytokine-induced expression of nitric oxide synthase in C2C12 skeletal muscle myocytes. Am. J. Physiol.

[95] Kobzik L., Stringer B., Balligand J.L., Reid M.B., Stamler J.S. (1995). Endothelial type nitric oxide synthase in skeletal muscle fibers: mitochondrial relationships. Biochem. Biophys. Res. Commun.

[96] Bates T.E., Loesch A., Burnstock G., Clark J.B. (1996). Mitochondrial nitric oxide synthase: A ubiquitous regulator of oxidative phosphorylation?. Biochem. Biophys. Res. Commun.

[97] Grozdanovic Z., Nakos G., Dahrmann G., Mayer B., Gossrau R. (1995). Species-independent expression of nitric oxide synthase in the sarcolemma region of visceral and somatic striated muscle fibers. Cell Tissue Res.

[98] Heinzel B., John M., Klatt P., Bohme E., Mayer B. (1992). Ca^2+^/calmodulin-dependent formation of hydrogen peroxide by brain nitric oxide synthase. Biochem. J.

[99] Laursen J.B., Somers M., Kurz S., McCann L., Warnholtz A., Freeman B.A., Tarpey M., Fukai T., Harrison D.G. (2001). Endothelial regulation of vasomotion in apoE-deficient mice: Implications for interactions between peroxynitrite and tetrahydrobiopterin. Circulation.

[100] Cai H., Harrison D.G. (2000). Endothelial dysfunction in cardiovascular diseases: The role of oxidant stress. Circ. Res.

[101] Xia Y., Tsai A.L., Berka V., Zweier J.L. (1998). Superoxide generation from endothelial nitric-oxide synthase. A Ca^2+^/calmodulin-dependent and tetrahydrobiopterin regulatory process. J. Biol. Chem.

[102] Vasquez-Vivar J., Kalyanaraman B., Martasek P., Hogg N., Masters B.S., Karoui H., Tordo P., Pritchard K.A. (1998). Superoxide generation by endothelial nitric oxide synthase: The influence of cofactors. Proc. Natl. Acad. Sci. USA.

[103] Wink D.A., Cook J.A., Pacelli R., Liebmann J., Krishna M.C., Mitchell J.B. (1995). Nitric oxide (NO) protects against cellular damage by reactive oxygen species. Toxicol. Lett..

[104] Brenman J.E., Chao D.S., Gee S.H., McGee A.W., Craven S.E., Santillano D.R., Wu Z., Huang F., Xia H., Peters M.F. (1996). Interaction of nitric oxide synthase with the postsynaptic density protein PSD-95 and alpha1-syntrophin mediated by PDZ domains. Cell.

[105] Rando T.A. (2001). Role of nitric oxide in the pathogenesis of muscular dystrophies: A “two hit” hypothesis of the cause of muscle necrosis. Microsc. Res. Tech.

[106] Carmignac V., Durbeej M. (2012). Cell-matrix interactions in muscle disease. J. Pathol.

[107] Van Deutekom J.C., van Ommen G.J. (2003). Advances in Duchenne muscular dystrophy gene therapy. Nat. Rev. Genet.

[108] Brenman J.E., Chao D.S., Xia H., Aldape K., Bredt D.S. (1995). Nitric oxide synthase complexed with dystrophin and absent from skeletal muscle sarcolemma in Duchenne muscular dystrophy. Cell.

[109] Lai Y., Thomas G.D., Yue Y., Yang H.T., Li D., Long C., Judge L., Bostick B., Chamberlain J.S., Terjung R.L. (2009). Dystrophins carrying spectrin-like repeats 16 and 17 anchor nNOS to the sarcolemma and enhance exercise performance in a mouse model of muscular dystrophy. J. Clin. Invest.

[110] Lai Y., Zhao J., Yue Y., Duan D. (2013). α2 and α3 helices of dystrophin R16 and R17 frame a microdomain in the α1 helix of dystrophin R17 for neuronal NOS binding. Proc. Natl. Acad. Sci. USA.

[111] Wehling-Henricks M., Tidball J.G. (2011). Neuronal nitric oxide synthase-rescue of dystrophin/utrophin double knockout mice does not require nNOS localization to the cell membrane. PLoS One.

[112] Keesey J.C. (2004). Clinical evaluation and management of myasthenia gravis. Muscle Nerve.

[113] Meinen S., Lin S., Ruegg M.A., Punga A.R. (2012). Fatigue and muscle atrophy in a mouse model of myasthenia gravis is paralleled by loss of sarcolemmal nNOS. PLoS One.

[114] Berchtold M.W., Brinkmeier H., Muntener M. (2000). Calcium ion in skeletal muscle: Its crucial role for muscle function, plasticity, and disease. Physiol. Rev.

[115] Grozdanovic Z., Baumgarten H.G. (1999). Nitric oxide synthase in skeletal muscle fibers: A signaling component of the dystrophin-glycoprotein complex. Histol. Histopathol.

[116] Beltman J.G., de Haan A., Haan H., Gerrits H.L., van Mechelen W., Sargeant A.J. (2004). Metabolically assessed muscle fibre recruitment in brief isometric contractions at different intensities. Eur. J. Appl. Physiol.

[117] Reid M.B. (1998). Role of nitric oxide in skeletal muscle: Synthesis, distribution and functional importance. Acta Physiol. Scand.

[118] Betzenhauser M.J., Marks A.R. (2010). Ryanodine receptor channelopathies. Pflugers Arch.

[119] Andersson D.C., Betzenhauser M.J., Reiken S., Meli A.C., Umanskaya A., Xie W., Shiomi T., Zalk R., LaCampagne A., Marks A.R. (2011). Ryanodine receptor oxidation causes intracellular calcium leak and muscle weakness in aging. Cell Metab.

[120] Bellinger A.M., Reiken S., Dura M., Murphy P.W., Deng S.X., Landry D.W., Nieman D., Lehnart S.E., Samaru M., LaCampagne A. (2008). Remodeling of ryanodine receptor complex causes “leaky” channels: A molecular mechanism for decreased exercise capacity. Proc. Natl. Acad. Sci. USA.

[121] Gehlert S., Bungartz G., Willkomm L., Korkmaz Y., Pfannkuche K., Schiffer T., Bloch W., Suhr F. (2012). Intense resistance exercise induces early and transient increases in ryanodine receptor 1 phosphorylation in human skeletal muscle. PLoS One.

[122] Bellinger A.M., Mongillo M., Marks A.R. (2008). Stressed out: The skeletal muscle ryanodine receptor as a target of stress. J. Clin. Invest.

[123] Bellinger A.M., Reiken S., Carlson C., Mongillo M., Liu X., Rothman L., Matecki S., LaCampagne A., Marks A.R. (2009). Hypernitrosylated ryanodine receptor calcium release channels are leaky in dystrophic muscle. Nat. Med.

[124] Sun J., Xin C., Eu J.P., Stamler J.S., Meissner G. (2001). Cysteine-3635 is responsible for skeletal muscle ryanodine receptor modulation by NO. Proc. Natl. Acad. Sci. USA.

[125] Suhr F., Gehlert S., Braun K., Bungartz G., Kern P., Willkomm L., Pfannkuche K., Krüger M., Bloch W (2013).

[126] Shen W., Hintze T.H., Wolin M.S. (1995). Nitric oxide. An important signaling mechanism between vascular endothelium and parenchymal cells in the regulation of oxygen consumption. Circulation.

[127] Morrison R.J., Miller C.C., Reid M.B. (1996). Nitric oxide effects on shortening velocity and power production in the rat diaphragm. J. Appl. Physiol..

[128] Perkins W.J., Han Y.S., Sieck G.C. (1997). Skeletal muscle force and actomyosin ATPase activity reduced by nitric oxide donor. J. Appl. Physiol.

[129] Marechal G., Beckers-Bleukx G. (1998). Effect of nitric oxide on the maximal velocity of shortening of a mouse skeletal muscle. Pflugers Arch.

[130] Morrison R.J., Miller C.C., Reid M.B. (1998). Nitric oxide effects on force-velocity characteristics of the rat diaphragm. Comp. Biochem. Physiol. A.

[131] Evangelista A.M., Rao V.S., Filo A.R., Marozkina N.V., Doctor A., Jones D.R., Gaston B., Guilford W.H. (2010). Direct regulation of striated muscle myosins by nitric oxide and endogenous nitrosothiols. PLoS One.

[132] Halliwell B., Cross C.E. (1994). Oxygen-derived species: Their relation to human disease and environmental stress. Environ. Health Perspect.

[133] Vollaard N.B., Shearman J.P., Cooper C.E. (2005). Exercise-induced oxidative stress: Myths, realities and physiological relevance. Sports Med.

[134] Boveris A., Chance B. (1973). The mitochondrial generation of hydrogen peroxide. General properties and effect of hyperbaric oxygen. Biochem. J.

[135] Xia R., Webb J.A., Gnall L.L., Cutler K., Abramson J.J. (2003). Skeletal muscle sarcoplasmic reticulum contains a NADH-dependent oxidase that generates superoxide. Am. J. Physiol Cell Physiol.

[136] Espinosa A., Leiva A., Pena M., Muller M., Debandi A., Hidalgo C., Carrasco M.A., Jaimovich E. (2006). Myotube depolarization generates reactive oxygen species through NAD(P)H oxidase; ROS-elicited Ca^2+^ stimulates ERK, CREB, early genes. J. Cell Physiol.

[137] Hidalgo C., Sanchez G., Barrientos G., Aracena-Parks P. (2006). A transverse tubule NADPH oxidase activity stimulates calcium release from isolated triads via ryanodine receptor type 1 *S*-glutathionylation. J. Biol. Chem.

[138] Sen C.K., Packer L. (1996). Antioxidant and redox regulation of gene transcription. FASEB J.

[139] Reid M.B. (2001). Invited review: Redox modulation of skeletal muscle contraction: What we know and what we don’t. J. Appl. Physiol.

[140] Supinski G.S., Callahan L.A. (2007). Free radical-mediated skeletal muscle dysfunction in inflammatory conditions. J. Appl. Physiol.

[141] Reid M.B., Khawli F.A., Moody M.R. (1993). Reactive oxygen in skeletal muscle. III. Contractility of unfatigued muscle. J. Appl. Physiol.

[142] Ji L.L., Stratman F.W., Lardy H.A. (1988). Antioxidant enzyme systems in rat liver and skeletal muscle. Influences of selenium deficiency, chronic training, and acute exercise. Arch. Biochem. Biophys.

[143] Powers S.K., Criswell D., Lawler J., Ji L.L., Martin D., Herb R.A., Dudley G. (1994). Influence of exercise and fiber type on antioxidant enzyme activity in rat skeletal muscle. Am. J. Physiol.

[144] Criswell D., Powers S., Dodd S., Lawler J., Edwards W., Renshler K., Grinton S. (1993). High intensity training-induced changes in skeletal muscle antioxidant enzyme activity. Med. Sci. Sports Exerc.

[145] Lawler J.M., Kwak H.B., Song W., Parker J.L. (2006). Exercise training reverses downregulation of HSP70 and antioxidant enzymes in porcine skeletal muscle after chronic coronary artery occlusion. Am. J. Physiol. Regul. Integr. Comp. Physiol.

[146] Bjornstedt M., Xue J., Huang W., Akesson B., Holmgren A. (1994). The thioredoxin and glutaredoxin systems are efficient electron donors to human plasma glutathione peroxidase. J. Biol. Chem.

[147] Bjornstedt M., Kumar S., Bjorkhem L., Spyrou G., Holmgren A. (1997). Selenium and the thioredoxin and glutaredoxin systems. Biomed. Environ. Sci.

[148] Lawler J.M., Powers S.K., Van D.H., Visser T., Kordus M.J., Ji L.L. (1994). Metabolic and antioxidant enzyme activities in the diaphragm: Effects of acute exercise. Respir. Physiol.

[149] Karanth J., Kumar R., Jeevaratnam K. (2004). Response of antioxidant system in rats to dietary fat and physical activity. Indian J. Physiol. Pharmacol.

[150] Lambertucci R.H., Levada-Pires A.C., Rossoni L.V., Curi R., Pithon-Curi T.C. (2007). Effects of aerobic exercise training on antioxidant enzyme activities and mRNA levels in soleus muscle from young and aged rats. Mech. Ageing Dev.

[151] Leeuwenburgh C., Fiebig R., Chandwaney R., Ji L.L. (1994). Aging and exercise training in skeletal muscle: Responses of glutathione and antioxidant enzyme systems. Am. J. Physiol.

[152] Vincent H.K., Powers S.K., Stewart D.J., Demirel H.A., Shanely R.A., Naito H. (2000). Short-term exercise training improves diaphragm antioxidant capacity and endurance. Eur. J. Appl. Physiol.

[153] Tibballs J. (1993). The role of nitric oxide (formerly endothelium-derived relaxing factor-EDRF) in vasodilatation and vasodilator therapy. Anaesth. Intensive Care.

[154] Hirai D.M., Copp S.W., Ferguson S.K., Holdsworth C.T., McCullough D.J., Behnke B.J., Musch T.I., Poole D.C. (2012). Exercise training and muscle microvascular oxygenation: Functional role of nitric oxide. J. Appl. Physiol.

[155] Ferguson S.K., Hirai D.M., Copp S.W., Holdsworth C.T., Allen J.D., Jones A.M., Musch T.I., Poole D.C. (2013). Impact of dietary nitrate supplementation via beetroot juice on exercising muscle vascular control in rats. J. Physiol.

[156] Wolosker H., Panizzutti R., Engelender S. (1996). Inhibition of creatine kinase by *S*-nitrosoglutathione. FEBS Lett.

[157] Gross W.L., Bak M.I., Ingwall J.S., Arstall M.A., Smith T.W., Balligand J.L., Kelly R.A. (1996). Nitric oxide inhibits creatine kinase and regulates rat heart contractile reserve. Proc. Natl. Acad. Sci. USA.

[158] Wilson C.M., Cushman S.W. (1994). Insulin stimulation of glucose transport activity in rat skeletal muscle: Increase in cell surface GLUT4 as assessed by photolabelling. Biochem. J.

[159] Youn J.H., Gulve E.A., Holloszy J.O. (1991). Calcium stimulates glucose transport in skeletal muscle by a pathway independent of contraction. Am. J. Physiol.

[160] Lund S., Holman G.D., Schmitz O., Pedersen O. (1995). Contraction stimulates translocation of glucose transporter GLUT4 in skeletal muscle through a mechanism distinct from that of insulin. Proc. Natl. Acad. Sci. USA.

[161] Roy D., Marette A. (1996). Exercise induces the translocation of GLUT4 to transverse tubules from an intracellular pool in rat skeletal muscle. Biochem. Biophys. Res. Commun.

[162] Lemieux K., Konrad D., Klip A., Marette A. (2003). The AMP-activated protein kinase activator AICAR does not induce GLUT4 translocation to transverse tubules but stimulates glucose uptake and p38 mitogen-activated protein kinases alpha and beta in skeletal muscle. FASEB J.

[163] Etgen G.J., Fryburg D.A., Gibbs E.M. (1997). Nitric oxide stimulates skeletal muscle glucose transport through a calcium/contraction- and phosphatidylinositol-3-kinase-independent pathway. Diabetes.

[164] Mohr S., Stamler J.S., Brune B. (1996). Posttranslational modification of glyceraldehyde-3-phosphate dehydrogenase by *S*-nitrosylation and subsequent NADH attachment. J. Biol. Chem.

[165] Brown G.C. (1995). Nitric oxide regulates mitochondrial respiration and cell functions by inhibiting cytochrome oxidase. FEBS Lett.

[166] Palacios-Callender M., Hollis V., Frakich N., Mateo J., Moncada S. (2007). Cytochrome c oxidase maintains mitochondrial respiration during partial inhibition by nitric oxide. J. Cell Sci.

[167] Glass D.J. (2003). Signalling pathways that mediate skeletal muscle hypertrophy and atrophy. Nat. Cell Biol.

[168] Sellman J.E., DeRuisseau K.C., Betters J.L., Lira V.A., Soltow Q.A., Selsby J.T., Criswell D.S. (2006). *In vivo* inhibition of nitric oxide synthase impairs upregulation of contractile protein mRNA in overloaded plantaris muscle. J. Appl. Physiol.

[169] Leiter J.R., Upadhaya R., Anderson J.E. (2012). Nitric oxide and voluntary exercise together promote quadriceps hypertrophy and increase vascular density in female 18-mo-old mice. Am. J. Physiol. Cell Physiol.

[170] Ito N., Ruegg U.T., Kudo A., Miyagoe-Suzuki Y., Takeda S. (2013). Activation of calcium signaling through Trpv1 by nNOS and peroxynitrite as a key trigger of skeletal muscle hypertrophy. Nat. Med.

[171] Samengo G., Avik A., Fedor B., Whittaker D., Myung K.H., Wehling-Henricks M., Tidball J.G. (2012). Age-related loss of nitric oxide synthase in skeletal muscle causes reductions in calpain *S*-nitrosylation that increase myofibril degradation and sarcopenia. Aging Cell.

[172] Gundersen K., Bruusgaard J.C. (2008). Nuclear domains during muscle atrophy: Nuclei lost or paradigm lost?. J. Physiol.

[173] Qaisar R., Renaud G., Morine K., Barton E.R., Sweeney H.L., Larsson L. (2012). Is functional hypertrophy and specific force coupled with the addition of myonuclei at the single muscle fiber level?. FASEB J.

[174] Walker D.K., Fry C.S., Drummond M.J., Dickinson J.M., Timmerman K.L., Gundermann D.M., Jennings K., Volpi E., Rasmussen B.B. (2012). PAX7+ satellite cells in young and older adults following resistance exercise. Muscle Nerve.

[175] Mackey A.L., Esmarck B., Kadi F., Koskinen S.O., Kongsgaard M., Sylvestersen A., Hansen J.J., Larsen G., Kjaer M. (2007). Enhanced satellite cell proliferation with resistance training in elderly men and women. Scand. J. Med. Sci. Sports.

[176] Martins K.J., St-Louis M., Murdoch G.K., MacLean I.M., McDonald P., Dixon W.T., Putman C.T., Michel R.N. (2012). Nitric oxide synthase inhibition prevents activity-induced calcineurin-NFATc1 signalling and fast-to-slow skeletal muscle fibre type conversions. J. Physiol.

[177] Zammit P.S., Partridge T.A., Yablonka-Reuveni Z. (2006). The skeletal muscle satellite cell: The stem cell that came in from the cold. J. Histochem. Cytochem.

[178] Friday B.B., Pavlath G.K. (2001). A calcineurin- and NFAT-dependent pathway regulates Myf5 gene expression in skeletal muscle reserve cells. J. Cell Sci.

[179] Tengan C.H., Rodrigues G.S., Godinho R.O. (2012). Nitric oxide in skeletal muscle: Role on mitochondrial biogenesis and function. Int. J. Mol. Sci.

[180] Cerqueira F.M., Laurindo F.R., Kowaltowski A.J. (2011). Mild mitochondrial uncoupling and calorie restriction increase fasting eNOS, akt and mitochondrial biogenesis. PLoS One.

[181] Hood D.A. (2009). Mechanisms of exercise-induced mitochondrial biogenesis in skeletal muscle. Appl. Physiol. Nutr. Metab.

[182] Hood D.A. (2001). Invited review: Contractile activity-induced mitochondrial biogenesis in skeletal muscle. J. Appl. Physiol.

[183] Gomes E.C., Silva A.N., de Oliveira M.R. (2012). Oxidants, antioxidants, and the beneficial roles of exercise-induced production of reactive species. Oxid. Med. Cell Longev.

[184] Jorgensen S.B., Richter E.A., Wojtaszewski J.F. (2006). Role of AMPK in skeletal muscle metabolic regulation and adaptation in relation to exercise. J. Physiol.

[185] Kulisz A., Chen N., Chandel N.S., Shao Z., Schumacker P.T. (2002). Mitochondrial ROS initiate phosphorylation of p38 MAP kinase during hypoxia in cardiomyocytes. Am. J. Physiol. Lung Cell Mol. Physiol.

[186] Liu C., Lin J.D. (2011). PGC-1 coactivators in the control of energy metabolism. Acta Biochim. Biophys. Sin. (Shanghai).

[187] Sriwijitkamol A., Coletta D.K., Wajcberg E., Balbontin G.B., Reyna S.M., Barrientes J., Eagan P.A., Jenkinson C.P., Cersosimo E., DeFronzo R.A. (2007). Effect of acute exercise on AMPK signaling in skeletal muscle of subjects with type 2 diabetes: A time-course and dose-response study. Diabetes.

[188] McConell G.K., Ng G.P., Phillips M., Ruan Z., Macaulay S.L., Wadley G.D. (2010). Central role of nitric oxide synthase in AICAR and caffeine-induced mitochondrial biogenesis in L6 myocytes. J. Appl. Physiol.

[189] Wadley G.D., Choate J., McConell G.K. (2007). NOS isoform-specific regulation of basal but not exercise-induced mitochondrial biogenesis in mouse skeletal muscle. J. Physiol.

[190] Clementi E., Nisoli E. (2005). Nitric oxide and mitochondrial biogenesis: A key to long-term regulation of cellular metabolism. Comp. Biochem. Physiol. A.

[191] Mortensen O.H., Frandsen L., Schjerling P., Nishimura E., Grunnet N. (2006). PGC-1alpha and PGC-1beta have both similar and distinct effects on myofiber switching toward an oxidative phenotype. Am. J. Physiol. Endocrinol. Metab.

[192] Gehlert S., Weber S., Weidmann B., Gutsche K., Platen P., Graf C., Kappes-Horn K., Bloch W. (2011). Cycling exercise-induced myofiber transitions in skeletal muscle depend on basal fiber type distribution. Eur. J. Appl. Physiol.

[193] Murgia M., Serrano A.L., Calabria E., Pallafacchina G., Lomo T., Schiaffino S. (2000). Ras is involved in nerve-activity-dependent regulation of muscle genes. Nat. Cell Biol.

[194] Calabria E., Ciciliot S., Moretti I., Garcia M., Picard A., Dyar K.A., Pallafacchina G., Tothova J., Schiaffino S., Murgia M. (2009). NFAT isoforms control activity-dependent muscle fiber type specification. Proc. Natl. Acad. Sci. USA.

[195] Drenning J.A., Lira V.A., Simmons C.G., Soltow Q.A., Sellman J.E., Criswell D.S. (2008). Nitric oxide facilitates NFAT-dependent transcription in mouse myotubes. Am. J. Physiol. Cell Physiol.

